# Identification of Novel MET Exon 14 Skipping Variants in Non-Small Cell Lung Cancer Patients: A Prototype Workflow Involving in Silico Prediction and RT-PCR

**DOI:** 10.3390/cancers14194814

**Published:** 2022-10-01

**Authors:** Riku Das, Maureen A. Jakubowski, Jessica Spildener, Yu-Wei Cheng

**Affiliations:** Department of Laboratory Medicine, Robert J. Tomsich Pathology and Laboratory Medicine Institute, Cleveland Clinic, 9500 Euclid Avenue, Cleveland, OH 44195, USA

**Keywords:** MET proto-oncogene, non-canonical splicing site, exon skipping, next-generation sequencing (NGS), non-small cell lung cancer (NSCLC), in silico prediction

## Abstract

**Highlights:**

MET exon 14 skipping is an oncogenic targetable driver mutation in lung cancer.

- Two novel non-canonical splice site variants identified in MET genome.
- Predicted splicing strength using in silico splicing prediction tools.
- Tested routine cytological smear slides for RNA-based molecular diagnostics.
- RT-PCR and Sanger sequencing analysis confirmed MET exon 14 skipping.

**Simple Summary:**

Non-small Cell Lung cancer (NSCLC) contributes to 85% of total lung cancer diagnoses in the United States. With the discovery of various targetable genetic markers and FDA approval of drugs against these markers, genetic testing has become a routine part of the diagnosis and staging process of NSCLC. MET gain of function mutations have been of particular interest as FDA has recently approved two MET inhibitors for the treatment of NSCLC patients with MET exon 14 skipping (METex14) mutations. However, an effective workflow for the classification of various METex14 mutations in the clinical testing laboratory has not been explored. In this report, we reveal two novel METex14 variants and propose a cost-effective and robust workflow for molecular diagnosis of MET variants contributing to exon 14 skipping with the use of readily available specimen sources.

**Abstract:**

Background and aims: The MET exon 14 skipping (METex14) is an oncogenic driver mutation that provides a therapeutic opportunity in non-small cell lung cancer (NSCLCs) patients. This event often results from sequence changes at the MET canonical splicing sites. We characterize two novel non-canonical splicing site variants of MET that produce METex14. Materials and Methods: Two variants were identified in three advanced-stage NSCLC patients in a next-generation sequencing panel. The potential impact on splicing was predicted using in silico tools. METex14 mutation was confirmed using reverse transcription (RT)-PCR and a Sanger sequencing analysis on RNA extracted from stained cytology smears. Results: The interrogated MET (RefSeq ID NM_000245.3) variants include a single nucleotide substitution, c.3028+3A>T, in intron 14 and a deletion mutation, c.3012_3028del, in exon 14. The in silico prediction analysis exhibited reduced splicing strength in both variants compared with the MET normal transcript. The RT-PCR and subsequent Sanger sequencing analyses confirmed METex14 skipping in all three patients carrying these variants. Conclusion: This study reveals two non-canonical MET splice variants that cause exon 14 skipping, concurrently also proposes a clinical workflow for the classification of such non-canonical splicing site variants detected by routine DNA-based NGS test. It shows the usefulness of in silico prediction to identify potential METex14 driver mutation and exemplifies the opportunity of routine cytology slides for RNA-based testing.

## 1. Introduction

Lung cancer is the leading cause of cancer death in the United States, with non-small cell lung cancer (NSCLC) contributing to 85% of total lung cancer diagnoses [1]. NSCLC patients with driver mutation who receive the appropriate targeted therapy have shown improved outcomes [2,3]. Among the actionable mutations for NSCLC treatment, mutations in EGFR, BRAF, KRAS, NTRK1/2/3, and ALK and ROS1 rearrangements are worth mentioning. Recently, the FDA has approved two drugs, capmatinib and tepotinib, for metastatic NSCLC with MET exon 14 skipping (METex14) mutation [3,4,5,6,7]. Upon treatment with MET tyrosin kinase inhibitor, patients with METex14 stage IV NSCLC survived longer. METex14 is observed in 3 to 4% of total NSCLC adenocarcinomas, the prevalence of which is greater or equal to some of the other oncogenic driver mutations: ROS1 (1–2%), NTRK1/2/3 (<1%), and BRAF (1–5%) [2,3,5,8,9,10,11,12].

MET proto-oncogene is located at chromosome 7q21-q31, which encodes for a receptor tyrosine kinase, c-Met, and is activated by ligand hepatocyte growth factor (HGF). Upon activation, MET phosphorylates its substrate and results in the activation of multiple signaling pathways (PI3K-AKT-mTOR, RAS-RAF-MEK-ERK, and FAK) leading to cell growth, proliferation, survival, adhesion, migration, and differentiation [13]. MET gain-of-function mutation has been recognized as a primary oncogenic driver that contributes to resistance towards many tyrosine kinase inhibitors in NSCLC treatment. Various MET gene alterations that lead to gain-of-function are sequence changes at MET exon 14 and flanking intronic regions, MET gene amplification, and MET gene fusions. Among them, METex14 is the most widely reported, 4–40% of which can occur concurrently with MET amplification [3,14,15]. However, other mechanisms of increased MET expression also play an important role in tumorigenesis driven by c-Met [16].

MET gene exon 14 encodes for a regulatory site in the juxtamembrane domain of c-Met protein. This site bears the binding site of Cbl, an E3 ubiquitin ligase, which leads to c-Met degradation upon binding [17]. Therefore, any alterations that cause exon 14 skipping leads to enhanced c-Met signaling and oncogenic transformation [18,19]. These alterations on the DNA level could be within the exon 14 (Y1003X or D1010X), in the intronic region surrounding the exon 14, or the total deletion of exon 14. Interestingly, the majority of these reported alterations are either partially deleted exon 14, or disruptions of the canonical splicing acceptor (AG) or donor (GT) sites of MET intron 13 and intron 14, respectively. However, the impact on METex14 caused by MET variants not involving the intron 14 canonical splicing donor site has seldom been addressed. With the approval of c-Met targeted drugs, identifying and accurately interpreting MET variants that increase c-Met signaling is of great targeted therapeutic importance. In this report, we describe a prototype workflow using in silico splicing prediction tools to identify MET variants of potential impact on the exon 14 splicing, followed by an RT-PCR and Sanger sequencing to confirm the splicing event, with a special focus on two novel MET variants located near the exon 14 and intron 14 juncture, but which do not disrupt the intron 14 canonical splicing site. Additionally, routine cytological smear slides were used to extract total RNA for the RT-PCR to determine the impact on METex14. Thereby, this study adds two novel variants to the growing list of METex14 variants [8,20,21] and demonstrates the utility of cytology slides as valuable sources for molecular diagnostic testing.

## 2. Methods

### 2.1. Sample Selection

With 3 years (2017 to 2019) of monitoring of the NSCLC specimens that were undergone in-house via the Cancer hotspot NGS test, we identified 20 cases of MET exon 14 and intron 14 genomic alterations. Out of the 20, we have identified three potential METex14 cases that do not involve canonical splicing sites. Two patients had novel variants identified in intron 14, c.3028+3A>T, and the third patient carried a variant in Exon 14, c.3012_3028del. These three NSCLC specimens were further investigated for the impact of MET exon 14 skipping at the RNA level. For the positive control, a patient’s specimen (cytology slides) with MET exon 14 canonical splicing donor site mutation that causes MET exon 14 skipping was used. For the negative control, an RNA specimen from a patient’s white blood cells without a history of NSCLC was used. The study was conducted according to the approved protocols of Cleveland Clinic’s Institutional Review Board (IRB; 17–177 and 19–329).

### 2.2. Patient Samples, DNA and RNA Extraction

Genomic DNA was extracted from the bronchial fluid of NSCLC patients, which was preserved in PreservCyt solution using the Maxwell RSC Cell DNA purification kit according to the manufacturer’s instruction (Promega, Madison, WI, USA). The quantity and quality of purified DNA were evaluated using Nanodrop and Qubit and stored at 4 °C until tested by Cancer hotspot NGS [22,23]. Direct smears were prepared from residual bronchial fluid, which were either diff-Quick or Papanicolaou (Pap)-stained. Selected diff-Quick and Pap-stained specimens were used for RNA extraction [24]. Total RNA was extracted using the Maxwell RSC RNA FFPE kit (Promega, Madison, WI, USA) from the smears to use in the RNA-based assay. The quantity and quality of total nucleic acid were evaluated using Nanodrop and Qubit and stored at −70 °C until tested.

### 2.3. Cancer Hotspot Panel Library Preparation, Sequencing, and Data Analysis

Cancer hotspot NGS library preparation was performed as described previously [22,23]. Briefly, 10 ng of genomic DNA and 207 PCR primers pairs (AmpliSeq Cancer Hotspot Panel v2.0 kit, Thermo Fisher Scientific, Waltham, MA, USA) were used for multiplex PCR to analyze approximately 2800 hotspot mutations in 50 genes. An oligonucleotide barcode was introduced into each sample to properly separate the sequencing reads of individual sample libraries. PCR amplicons were analyzed by Bioanalyzer 2100 for quality check and samples with >200 pM were pooled, followed by sequencing on the MiSeq instrument. The sequencing data were aligned to human genome build 19 (HG19/GRCh37) and variants in mutation hot spot regions in *BRAF*, *EGFR*, *ERBB2*, *KRAS*, and *MET* were identified using NextGENe Software (Soft Genetics, State College, PA, USA). The Integrative Genomics Viewer (IGV) was used to visually inspect the quality of read alignment and variant calls. A quality score of Q30 was used as filtering criteria to determine the sequence read quality. For a given sample, the minimum coverage requirement of targeted regions was 100×. Variants with variant allele frequencies (VAFs) as low as 2% may be identified using this method. The MET RefSeq transcript NM_000245.3 is used for variant data analysis and reporting.

### 2.4. In Silico Prediction

In silico splice tools, including SpliceSiteFinder-like, MaxEntScan, NNSplice, and GeneSplicer, were integrated in the Alamut Visual Plus (Version 1.3, SOPHiA GENETICS, Lausanne, Switzerland) for the prediction of the MET variant’s impact on gene splicing. In the Alamut Visual Plus, impacts on gene splicing from individual tools are represented either with a vertical blue bar for 5′ donor sites or a vertical green bar for 3′ acceptor sites. Assigned scores, which are proportional to the heights of each bar, are indicators of splicing donor or acceptor signals that impact the splicing strength. Known constitutive signals are displayed as a small blue triangle for 5′ or a green triangle for 3′, close to the sequence letters.

### 2.5. RT-PCR and Sanger Sequencing

RNA specimens from the patients and negative control were reverse transcribed using the Ipsogen Reverse Transcription kit (Qiagen, Hilden, Germany) with random primers. The obtained cDNA was amplified using a forward primer specific to MET Exon 13, 5′-GCTGGTGTTGTCTCAATATCAA-3′ and a reverse primer specific to MET Exon 15, 5′-GGCATGAACCGTTCTGAGAT-3′. The PCR conditions are as follows: 95 °C for 3 min and 45 cycles of 95 °C for 30 s, 55 °C for 3 s, and 72 °C for 2 min. The PCR products were analyzed using Bioanalyzer and the splicing products were subjected to Sanger sequencing. Sanger sequencing was performed using a modified protocol supplied by Applied Biosystems BigDye Terminator 1.1 and 3.1 Cycle sequencing kits. Fragments were then analyzed using Applied Biosystems 48-capillary 3730 Genomic Analyzer.

## 3. Results

### 3.1. Demographic and Clinical Characteristics of NSCLC Patients with Two Novel MET Variants

**Figure 1:**
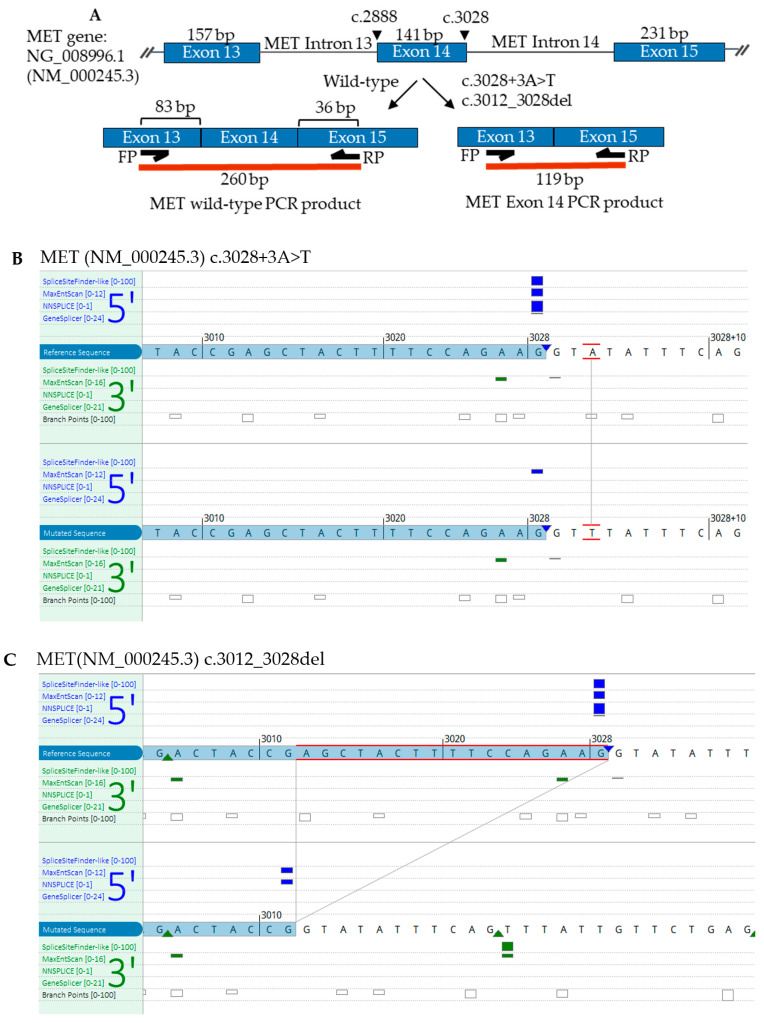
(**A**) Graphic representation of MET exon 13, 14, and 15 sizes, primers for RT-PCR binding sites, and predicted size of wild-type and MET exon 14 PCR products. (**B**,**C**) Alamut visual prediction of MET splicing with c.3028+3A>T and c.3012_3028del variants. With the use of four splicing predictors, donor prediction signals, shown with vertical blue bars (each bar corresponded to an individual splicing predictor) at the 5′ donor sites are reduced, suggesting that these variants alter splicing. Vertical green bars are for 3′ acceptor sites showing minimum or no change. Heights of the bars are proportional to splicing strength. Known constitutive signals are displayed as a small blue triangle for 5′ or a green triangle for 3′.

Three advanced-stage NSCLC patients with one exon 14 and the other intron 14 novel MET variants were identified from an in-house lung cancer NGS test. Patients’ demography and clinical characteristics are shown in Table 1. Patient 1 and Patient 2 harbored the same MET single nucleotide variant c.3028+3A>T at the beginning of intron 14, with 24% and 37% allelic fractions, respectively. This variant was near but not at the canonical splice donor sequence (Figure 1A,B). Patient 3 carried another rare MET variant with a 17-nucleotide deletion at the 3′ end of the exon 14, c.3012_3028delAGCTACTTTTCCAGAAG, with 11% allelic fraction (Figure 1A,C). In all cases, variants were identified with very high numbers of sequencing coverages (Table 1). Patients 1 and 3 did not carry other actionable mutations in BRAF, EGFR, HER2, KRAS, and ALK rearrangement. Patient 2 had a mutation in KRAS (NM_004985.3 c.34G>T, p.Gly12Cys) with 10% allelic fraction.

Since the MET variants were identified near the exon–intron junction, we performed an in silico analysis for possible impact in splicing. Using the splicing prediction tool analysis, we have observed a drastic reduction in splicing strength at the MET intron 14 splicing donor site in both variants (c.3028+3A>T and c.3012_3028del), compared with the MET wild-type transcript (Figure 1B,C), suggesting the possibility of splicing alterations leading to the exon 14 skipping.

### 3.2. Confirmation of MET Exon 14 Mutation in Two Novel MET Variants

**Figure 2:**
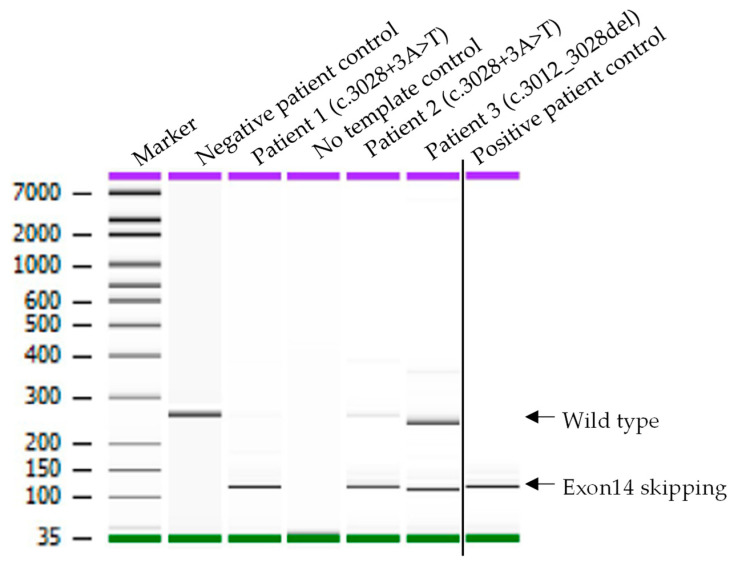
RT-PCR analysis on RNA from negative control and four patients with suspected (Patient 1 to 3) or known (positive control) METEx14 skipping variant, bronchial fine needle aspirate specimens. Gel picture from Bioanalyzer showing PCR products amplified using specific primers (MET_FP1 and MET_RP1). A fragment size of 260 indicates MET WT, and a fragment of 119 bp in size indicates MET exon 14 variant. No variant was identified in negative leukocytes.

**Figure 3:**
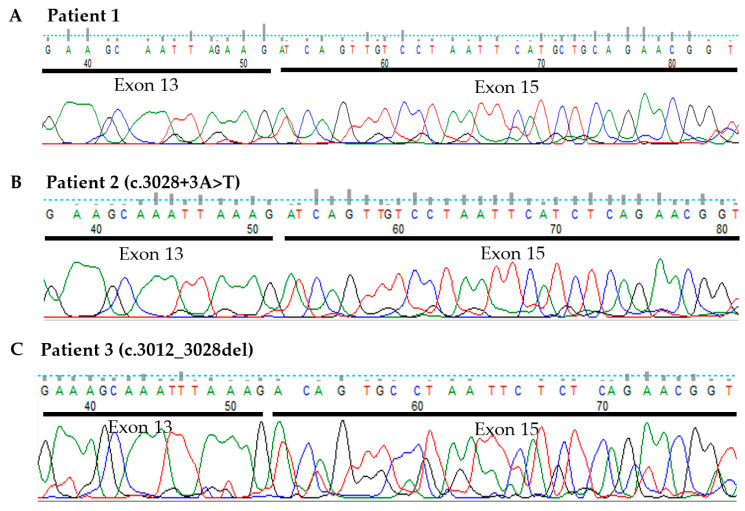
Sanger sequencing on PCR products obtained from Bioanalyzer polyacrylamide gel. Exon 13 and 15 sequences are indicated with black bars. (**A**) Negative patient control with 260 bp PCR product. (**B**) Patient 1 with MET c.3028+3A>T variant, 119 bp product. (**C**) Patient 1 with MET c.3028+3A>T variant, 119 bp product. Patient 3 with MET c.3012_3028del, 119 bp product.

To provide functional evidence of these two MET variants causing a splicing defect and exon 14 skipping, an RT-PCR and Sanger sequencing analysis were performed. The positions of PCR primers and predicted amplicon sizes for MET wild-type (WT) and METex14 are shown in Figure 1A. The primers were designed—with forward binding to exon 13 and reverse to exon 15—and estimated to produce 260 bp wild-type (without exon 14 skipping) or 119 bp METex14 amplicons. RNA isolated from the diff-Quick smears were reverse transcribed to cDNA using random primers, followed by the amplification of cDNA with the gene-specific primers. As shown in Figure 2, the negative patient control produced a single fragment of approximately 260 bp in size, which matches with the calculated WT amplicon size. However, in addition to the WT PCR product, all three patients with MET variants and positive control produce a smaller fragment of roughly 119 bp in size, an expected METex14 product size. Of note, Patient 1 and Patient 2, as well as the positive control, showed a more robust amplification of the METex14 allele compared to the WT allele, whereas both alleles were somewhat equally amplified in Patient 3. A similar RT-PCR result of Patient 1 was also observed using RNA extracted from a Pap-stained slide (data not shown). Sequencing of the three patients’ 119 bp PCR products revealed the splicing junction spanning the last nucleotide of exon 13 and the first nucleotide of exon 15 with the total omission of the exon 14 sequence (Figure 3A–C). Sequencing of the 260 bp fragment from the negative patient control indeed showed MET WT amplicon with the sequence spanning the entire exon 14 sequence and portions of exon 13 and 15 (data not shown). Altogether, these data suggest that the two novel MET variants (c.3028+3A>T and c.3012_3028del) identified in the lung cancer panel cause exon 14 skipping in the MET transcript.

## 4. Discussion

**Figure 4:**
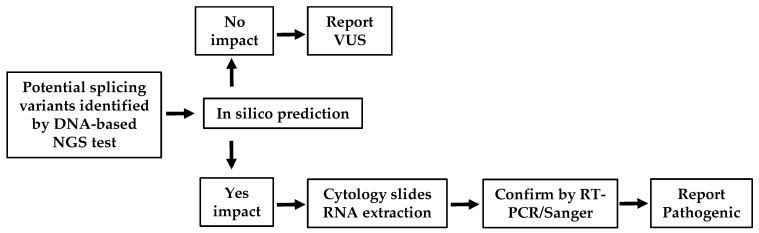
Model depicting a cost-effective clinical workflow to enable a potential genomic slicing variant screening process. When variants of uncertain significance around canonical splicing sites are identified in routine DNA-based NGS test, specimens can be assigned to a rapid in silico analysis to identify their impact in mRNA splicing. A variant can be reported as VUS if no impact is found in in silico prediction. If predictions suggest a significant impact in splicing, a specimen is assigned for an RNA work-up. The available cytology slides can be used to extract RNA, followed by RT-PCR and Sanger sequencing to convincingly identify splicing products.

MET mutations that produce MET gain-of-function have been growing in interest among clinicians for their use as an actionable oncogenic therapeutic target for NSCLC patients. Clinical trial data, based on which the first MET-targeted therapy was approved in 2020, indicated that NSCLC patients with METex14 somatic mutation show better outcomes with longer survival [3,6,7,25,26,27]. In this report, for the benefit of NSCLC patient management, we demonstrate a cost-effective and robust workflow (Figure 4) to definitively determine MET variants that contribute to exon 14 skipping.

It is well known that the canonical splice donor GT and acceptor AG dinucleotide sites are required for spliceosome interaction and subsequent splicing of the intronic sequences in pre-mRNA. Thus, in a molecular diagnostic laboratory, variants identified at the canonical splice sites are mostly classified as likely pathogenic (LP) or pathogenic due to the well-established biological impacts on gene splicing. Evidence also suggests that the immediate vicinity of 12–30 and 15–33 nucleotides surrounding the intronic donor and acceptor site, respectively, may contribute to the splicing efficiency by proving a preferential low folding strength [28]. In addition, splicing signals are also present in the exons, which are either called exonic splicing enhancer (ESE) to facilitate the splicing, or exonic splicing suppressor (ESS) to suppress splicing. These are located close to the splicing donor or acceptor sites and serve as binding sites of Ser/Arg-rich proteins (SR proteins) through their RNA-binding domain that help multiple steps of the splicing pathway, including the recruitment of spliceosome to the exon–intron junctions. The importance of these sites was previously widely validated in the mutational analysis experiments [29,30,31,32], as well as a computational method [33,34]. However, the impacts of non-canonical splice site variants on gene splicing remain investigational and rely on bioinformatic prediction tools to identify any potential candidates. Additionally, according to the Standards and Guidelines for the Interpretation of Sequence Variants issued by the American College of Medical Genetics and Genomics and the Association for Molecular Pathology, computational evidence predicting a deleterious effect is not sufficient to promote the identified variant from a variant of unknown significance (VUS) to the LP category without functional data [35].

To our knowledge, the presence of the c.3028+3A>T variant was not reported previously, as searched in the COSMIC, cBioPortal or in the population database, gnomAD. Rather, another variant was reported at the same nucleotide position (c.3028+3A>G) in a patient with pulmonary sarcomatoid carcinoma, which led to MET exon 14 skipping [36]. Consequently, when the MET c.3028+3A>T variant was identified in our clinical laboratory, it was classified as VUS because of the lack of direct evidence to meet the LP classification criteria, while reported with a caveat alongside the VUS classification, suggesting the likelihood of the c.3028+3A>T variant’s contribution to MET exon 14 skipping. However, for the best practice of a molecular diagnostic laboratory, it is important to issue a report with a definitive test result as well as interpretation to avoid miscommunication between the testing laboratories and the caring clinicians. It is of interest to mention Patient 3, who carries the c.3012_3028del variant and is predicted to be a gain-of-function mutation. This patient has no other disease-causing variants in the hotspot regions of BRAF, EGFR, ERBB2, KRAS, and has no genomic rearrangements in the ALK and ROS1 genes. More importantly, the patient’s condition was dramatically improved in just 8 weeks of treatment with crizotinib, even though this particular variant has not been reported in the somatic cancer databases. Our study now shows that it has a drastic impact on exon 14 splicing. Furthermore, a substitution mutation at c.3028G>A has shown the disruption of the splice donor site causing METex14 skipping [8]. The same study also demonstrated that the genomic deletion involving MET c.3028 and the canonical intron 14 splicing donor site (e.g., c.3010_3028+8del, c.3018_3028+8del, c.3020_3028+24del) accounts for 61% of MET exon 14 skipping mutations [8]. Altogether, our data and others indicate the importance of nucleotide position c.3028 and the surrounding sequences in regulating the MET exon 14 splicing event.

We have observed in the RT-PCR results that WT transcript levels are not proportional to the allelic fractions observed in the DNA-NGS analysis in Patients 1 and 2. WT transcripts were either near the detection limit (Patient 1) or at reduced levels (Patient 2), even when the variant allelic fractions were well below 50%. These observations are likely attributed to either (1) an inaccurate estimation of tumor cells in each specimen; (2) the non-quantification nature of the end-point PCR test; (3) the uneven distribution of the tumor and infiltrating stromal cells in the process of making various types of specimens for different downstream analyses. For the NGS analysis, paraffin blocks were used, whereas cell-smeared slides (Diff-Quick and pap-stained) were used for the RT-PCR assay. Even two smear slides made from the same specimen source will not have equal proportions of normal and malignant cells due to uncontrolled cell separation during the smearing preparation. A similar unproportioned transcript pattern was also seen in the previous reports of METex14 analysis in various RNA-based assays [8,21].

The ability of a diagnostic laboratory to determine the impact of a variant on gene splicing is essential. Our study warrants the importance of detecting actionable mutations with METex14 for NSCLC patients, including targets in MET exon 14 and surrounding introns. Additionally, METex14 detects better in an RNA-based NGS assay at a 4.2% rate compared to a 1.3% rate in a DNA-based NGS assay, which prompts clinicians to use a supplemental RNA-based panel [37,38]. However, the majority of molecular diagnostics laboratories use DNA-based NGS tests as a routine method to identify METex14 variants, which may not provide the proper functional evidence of exon 14 splicing. Here, we present a workflow (Figure 4) that facilitates the variant triage process to determine those potential candidates that require RT-PCR confirmation of the splicing products. The combination of in silico prediction, RT-PCR, and Sanger sequencing can be readily adopted to a laboratory standard operating procedure as a routine practice. It is worth noting that although fresh and frozen tissues are often the sample of choice for RNA-based techniques, there is a growing demand for the use of cytology samples that are already processed and stained for downstream molecular testing. The possibility of utilizing cytological slides in RNA-based diagnostic methods was previously validated using smeared and Giemsa or Diff-Quik stained slides [39]. In our study, cytology slides for the corresponding three patients were retrieved, and tissues from these slides were used for RNA extraction and the RT-PCR. The successful outcome of our procedure further affirms the possibility of incorporating cytology slides when other tissue sources are scarce in patients for the benefit of targeted lung cancer therapy.

## Figures and Tables

**Table 1:** Demographic and clinical characteristics.

Characteristics	Patient 1	Patient 2	Patient 3
Histology	Adenocarcinoma	Adenocarcinoma	Necrotic NSCLC
Tumor%	40	80	90
MET variant NG_008996.1 (NM_000245.3)	c.3028+3A>T VAF = 24% NGS read depth = 3761	c.3028+3A>T VAF = 37% NGS read depth = 10,184	c.3012_3028del VAF = 11% NGS read depth = 3518
Other activating mutations in hotspots of BRAF, EGFR, HER2, and KRAS	Negative	KRAS(NM_004985.3) c.34G>T (p.Gly12Cys)	Negative

Abbreviations, VAF = variant allele fraction.

## Data Availability

Data supporting the reported results can be obtained from the corresponding author.
